# Design and Validation of an Articulated Sensor Carrier to Improve the Automatic Pipeline Inspection

**DOI:** 10.3390/s19061394

**Published:** 2019-03-21

**Authors:** Antonio Ramirez-Martinez, Noé Amir Rodríguez-Olivares, Sergio Torres-Torres, Guillermo Ronquillo-Lomelí, Jorge Alberto Soto-Cajiga

**Affiliations:** 1 Department of Energy, Center for Engineering and Industrial Development (CIDESI), Santiago de Queretaro, Queretaro 76125, Mexico; sergio.torres@cidesi.edu.mx (S.T.-T.); gronquillo@cidesi.edu.mx (G.R.-L.); jsoto@cidesi.edu.mx (J.A.S.-C.); 2 Engineering department, Latin American Technological University Online (UTEL), Naucalpan de Juarez, Estado de Mexico 53370, Mexico

**Keywords:** pipeline inspection gauge, sensor carrier, stand-off, straight beam

## Abstract

Pipeline inspection gauges (PIGs) carry out automatic pipeline inspection with nondestructive testing (NDT) technologies like ultrasound, magnetic flux leakage, and eddy current. The ultrasonic straight beam allows technicians to determine the wall thickness of the pipeline through the time of flight diffraction (TOFD), providing the pipeline reconstruction and allowing the detection of several defects like dents or corrosion. If the pipeline is of a long distance, then the inspection process is automatic, and the fluid pressure pushes the PIG through the pipeline system. In this case, the PIG velocity and its axial alignment with the pipeline cannot be controlled. The PIG geometry, the pipeline deformations, and the girth welds cause a continuous chattering when the PIG is running, removing the transducers perpendicularity with the inspection points, which means that some echoes cannot be received. To reduce this problem, we propose a novel method to design a sensor carrier that takes into account the angularity and distance effects to acquire the straight beam echoes. The main advantage of our sensor carrier is that it can be used in concave and convex pipeline sections through geometric adjustments, which ensure that it is in contact with the inner pipe wall. Our improvement of the method is the characterization of the misalignment between the internal wall of the pipeline and the transducer. Later, we analyzed the conditions of the automatic pipeline inspection, the existing recommendations in state-of-the-art technology, and the different mechanical scenarios that may occur. For the mechanical design, we developed all the equations and rules. At the signal processing level, we set a fixed gain in the filtering step to obtain the echoes in a defined distance range without saturating the acquisition channels. For the validation, we compared through the mean squared error (MSE) our sensor carrier in a straight pipe section and a pipe elbow of steel versus other sensor carrier configurations. Finally, we present the design parameters for the development of the sensor carrier for different pipeline diameters.

## 1. Introduction

Carbon-steel pipelines are widely used to transport oil and water because they are the safest and most economical way to do it. These pipelines have defects and anomalies due to their wear from operation and from external conditions, which finally induce leaks. The leaks cause economical losses, ecological damage, and oil/water shortage for communities. The pipeline inspection gauges (PIGs) help to prevent these problems because they carry out the inspection with non-destructive technologies to ensure pipeline reliability [1]. The PIG runs inside the pipeline due to locational difficulties that could hinder the inspection through the external pipe wall [2]. The PIG is remotely controlled by an umbilical cord system when the pipeline is of a few hundred meters in length, and it has a closed-circuit television (CCTV) system to carry out visual inspections (VI) [3]. However, for long-distance pipeline systems, intelligent in-line inspection (ILI) PIGs carry out the inspection process automatically; in this case, the PIG is propelled by the fluid inside the pipeline and its velocity and axial alignment cannot be controlled. For long-distance pipeline systems, the PIG must be able to collect data with a circumferential resolution (cr) of 8 mm at every axial displacement of 3 mm, according to international standards [4,5]. The PIG calculates its axial displacement using odometers, gyroscopes, and externally installed markers, which signalize specific locations [6,7]. For long-distance pipeline systems, ultrasound, magnetic flux leakage, and eddy current are the most common technologies used for the automatic inspection [8,9,10].

Ultrasonic testing allows technicians to evaluate the volumetric reliability of the pipeline; it means that, with ultrasound signals, it is possible to localize and identify many discontinuities in materials that are both on and below the surface of the pipeline [11]. Many PIGs use the ultrasonic pulse-echo method to perform the automatic pipeline inspection because it provides wall thickness measurements with a greater accuracy than manual inspection or other techniques [12,13,14,15]. For all wall thicknesses, the technicians generate the virtual pipeline reconstruction, which allows them to detect many of the most common defects, such as cracks that occur parallel to the surfaces, as well as corrosion and discontinuities within the body of the pipeline (such as voids or porous areas) [16].

**Figure 1:**
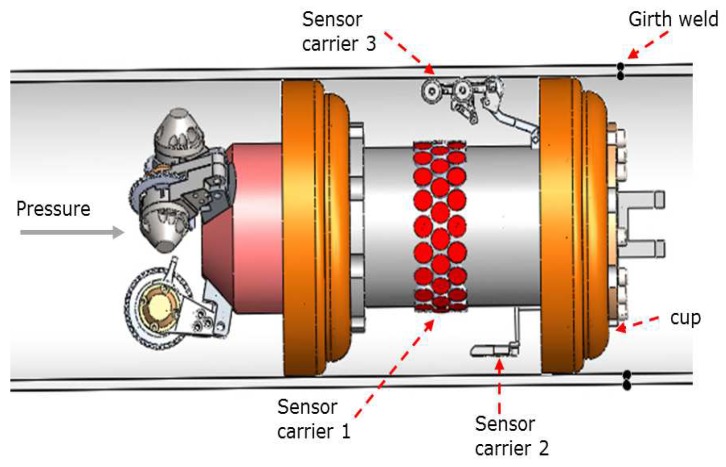
Pipeline inspection gauge capsule with different types of sensor carriers mounted.

The PIG geometry, the girth welds, and the pipeline deformations produce an axial vibration (chattering) in the PIG when it runs inside the pipeline [17,18]. This chattering is always present because there is a girth weld connection between two standard pipes at regular intervals of 12 m [17,19]. The chattering occurs as a result of the requirement for PIG cups to have tight contact with the inner pipe wall; this achieves the desired differential pressure across the pipeline, which overcomes the friction force and propels the PIG through the pipeline (see Figure 1) [20]. The chattering affects the wall thickness measurements because, if pulse-echo transducers (straight beam ultrasonic evaluation) are perpendicularly misaligned in relation to the inspection point, then the angle of incidence may be sufficiently large so as to miss the echoes. The problem of missing sample points of information increases when the PIG runs inside concave and convex pipe sections like pipe elbows [21] because they are the pipeline sections where corrosion is most likely to occur due to fluid stagnation [22]. In addition, the missing echoes affect the post-processing techniques applied to the thickness mesh construction (many post-processing techniques interpret each pipe wall thickness acquired as one pixel of an image) [12,23,24,25,26]. Therefore, the reliability of the fault detection falls until the detection capacity is not admissible (80% being the minimum acceptable value according to international standards [4,5]).

Larger diameter transducers could be considered to reduce the number of missing sample points caused by chattering, but they give an average thickness of an area larger than that covered by small diameter transducers, resulting in a lower resolution in the inspection [27]. In addition, with small diameter transducers, it is possible to reach the circumferential resolution of 8 mm without so many mechanical elements, which can result in an increased weight and size of the PIG. Some authors [27,28,29,30] have proposed new designs for PIGs, with the aim of reducing the chattering, protecting the PIG, and obtaining better measurements; however, these proposals are still under development. Another suggestion [31] is to increase the number of transducers in the area to be inspected, ensuring the correct measurement of the wall thickness; however, this action is relatively expensive, and it is only affordable for the oil industry and not for water distribution pipelines. It is important to mention that water pipeline inspection has become a necessity in recent years, so it is vital to reduce costs through the improvement of all PIG subsystems [32].

The specialized design of sensor carriers is the preferred way to reduce the loss of echoes caused by the chattering; these designs depend on the inspection requirements, and the configurations are different for wall thickness measurement, crack detection, combined inspection, and other particular tasks [33]. To improve the automatic wall thickness measurement; some sensor carriers [12,34] are installed in the PIG body (see Figure 1, sensor carrier 1); however, the distance between the transducers and the inner pipe wall complicates the measurement when the chattering is present and for certain sections of the curved pipeline [21]. Rigid sensor carriers [27,28,30,33] that bring the transducers as close as possible to the internal wall of the pipe are one of the best options for PIGs (see Figure 1, sensor carrier 2). However, when the PIG inspects sections of pipe that are not straight, it is difficult to maintain the perpendicular alignment with the transducers. One way to ensure tracking of the entire pipeline is the implementation of articulated sensor carriers that adapt to the shape of the trajectory (see Figure 1, sensor carrier 3). Geometrical PIGs [2,3,29] use these articulated sensor carriers with odometers in the wheels to reconstruct the trajectory of the PIG. Some authors have developed articulated sensor carriers for Eddy current and other inspection techniques [35,36], and they achieved excellent results using wheels that touch the inner pipe wall. However, each inspection technology has different considerations, and, in the case of ultrasonic straight beam testing, some authors [12,33] have mentioned some conditions: (a) Maintaining a distance of 20 mm between the piezoelectric transducer and the inner pipe wall (this distance is known as Stand-Off, denoted as $d_{SO}$) with water as a couplant; (b) Using piezoelectric transducers with a center frequency of 5 MHz; (c) Considering the ultrasonic signal not as an arrow, but a wavefront of acoustic energy.

Our goal is to develop a useful ultrasound system for automatic pipeline inspection. The electronic system has already been validated to measure thicknesses [37]. We use a specialized technique for online signal reduction [38], where the signals are stored in an *ad hoc* data storage system [39]. For the transducer excitation, we improved the ultrasonic pulse generator to maintain a fixed excitation voltage despite the chattering and the hammer effect [40]. However, all these improvements in the system are useless if it is not possible to ensure that the ultrasound signals capture. In this paper, we suggest an additional consideration for the sensor carrier design, which is the characterization of the misalignment between the inner pipe wall and the transducer. This is because, even with a small variation in angle, the amplitude and the number of echoes decreases. For the characterization, we also vary the $d_{SO}$ distance, finding the blind spot of the ultrasound beam. Our characterization does not reduce the blind spot; however, it allows us to establish a new $d_{SO}$ distance at which it was possible to obtain a better range of rotation to capture echoes. At the signal processing level, the characterization allowed us to propose a new gain *k* in the filtering process that improved the amplitude of the echoes without saturating the signals.

This paper presents a novel method to design a sensor carrier that ensures the angularity and distance to acquire the straight beam echoes. The sensor carrier improves the automatic pipeline wall thickness measure in concave and convex pipeline sections with piezoelectric ultrasound transducers. To achieve this, we added as the first step in the design method the characterization of the misalignment between the internal wall of the specimen and the transducer. In addition, we considered the conditions of the automatic pipeline inspection, the parameters of the ultrasonic straight beam, and the different scenarios that may occur during the inspection process. For the mechanical design, we developed all the equations and rules. At the signal processing level, we set a fixed gain *k* in the filtering step to compensate the sound pressure attenuation and to obtain the echoes in a defined distance range without saturating the acquisition channels. Thanks to the characterization step in the design method, it was possible to propose the diameter of the five wheels for the sensor carrier, with the view to protect the transducers and to maintain a fixed distance between the transducer and the inner pipe wall. The sensor carrier also has a set of springs which join together two blocks to ensure flexibility for concave and convex surfaces, and a connection link at the beginning of the sensor carrier to prevent the PIG getting jammed in the pipeline. The main advantages of our sensor carrier are: (a) An increase in detection capacity for straight beam echoes in concave and convex pipeline sections; (b) A reduction of the risk of the PIG getting lodged in the pipeline thanks to its mechanical configuration; (c) An unactuated system that does not consume energy from the battery system; (d) Self-adjustment for straight, concave and convex pipe surfaces; and (e) An increase in the margin of displacement between the transducer and the inner pipe wall, ensuring the echo capture despite of misalignment. Section 2 shows the theory for the ultrasonic pulse-echo immersion technique. Section 3 presents the characterization of the ultrasound beam for pipeline inspection and the mechanical sensor carrier design. Section 4 describes the results obtained with the proposed device. Section 5 outlines the discussion of the results and the parameters used to design the sensor carrier for other pipeline diameters. Finally, Section 6 shows the conclusions of this work.

## 2. Ultrasonic Pulse-Echo Immersion Technique

**Figure 2:**
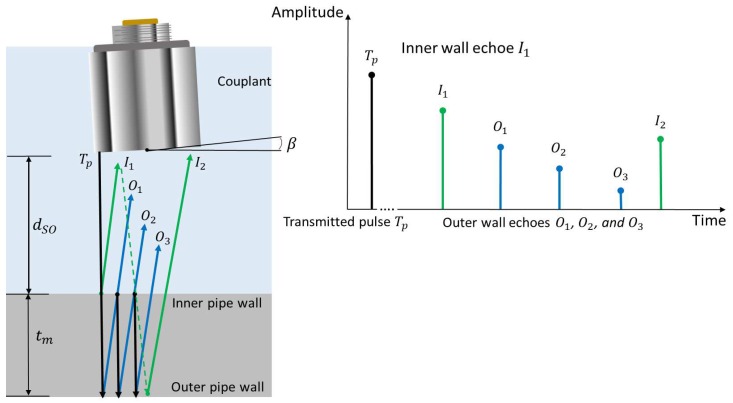
Physical parameters to ensure correct echo capture for the automatic pipeline inspection.

Several physical parameters must be considered (see Figure 2) to ensure the correct echo capture during automatic pipeline inspection: (a) The material to be inspected and its thickness (denoted as $t_{m}$)—in our case, the material is steel, and its width only decreases due to wear through use. (b) The couplant (oil or water) creates a stand-off, which is the distance (denoted as $d_{SO}$) between the transducer and the inner pipe wall to be inspected. The $d_{SO}$ distance causes a time delay that must be taken into account in the ultrasound acquisition system, and the delay length varies depending on the coupling medium, due to variances in the speed of sound in each medium. The existence of the $d_{SO}$ distance between the transducer and the inner pipe wall protects the transducer from wear, so this distance is beneficial. (c) The transducers are 5-MHz immersion piezoelectric. (d) The transmitted voltage pulse (denoted as $T_{p}$) ensures that the echoes will be received with enough energy to remove the noise generated by the high-frequency acquisition. (e) The misalignment angle (denoted as β) between the internal wall of the specimen and the transducer should ideally be zero; however, this condition can not always be guaranteed; therefore, it must be considered in the sensor carrier design as a parameter that exists in the automatic pipeline inspection. All these parameters allowed us to propose a novel method for the design of a sensor carrier that improves the detection capacity of straight beam echoes and facilitates the interpretation of information.

If the β angle is zero, and the $d_{SO}$ distance between the transducer and the inner pipe wall is correctly calibrated, then the echoes should have the order shown in Figure 2. The first echo ($T_{P}$) corresponds to the transmitted pulse, usually removed from the A-scan using a dead time; the second echo ($I_{1}$) corresponds to the internal wall, the time difference between the maximum of the echo transmitted ($T_{P}$) and the echo of the internal wall ($I_{1}$) determines the $d_{SO}$ distance between the transducer and the inner pipe wall; the third, fourth and fifth echoes ($O_{1}$, $O_{2}$ and $O_{3}$) are the bounces between the outer pipe wall and the inner pipe wall. The time difference between the echo $I_{1}$ and $O_{1}$ gives the wall thickness ($t_{m}$) of the pipeline, and the comparison of this value with the time difference between $O_{1}$ and $O_{2}$ helps to ensure the correct measurement. The echoes position are similar to Figure 2 when the $d_{SO}$ distance is constant and well adjusted. If the $d_{SO}$ distance changes or is not well fixed, then the echo positions change due to the different propagation velocities of the sound in the couplant and the steel, this change is in accordance with Equation (1):

(1) $$N=\frac{d_{SO}\;v_{m}}{t_{m}\;v_{c}}.$$

In Equation (1), $v_{c}$ is the acoustic velocity of the couplant (in m/s), $v_{m}$ is the acoustic velocity of the material to inspect (in m/s), and *N* is the number of echoes $O_{n}$ acquired between $I_{1}$ and $I_{2}$. For example, to inspect a 254 mm (10 inches) pipeline diameter of 1020-steel, with an acoustic velocity of 5890 m/s ($v_{s}$) and a wall thickness of 9.27 mm ($t_{m}$), with a $d_{SO}$ distance of 20 mm, in a water couplant with an acoustic velocity of 1480 m/s ($v_{c}$), then the *N* number of echoes to acquire between $I_{1}$ and $I_{2}$ is eight echoes. If the $d_{SO}$ distance changes, then the position of $I_{2}$ may move and appear in some position between $I_{1}$ and $O_{3}$, complicating the wall thickness measurement. In addition, if the β angle deviates from zero degrees, then Equation (1) is no longer valid because of the wavefront behavior of acoustic energy. The changes in the β angle and $d_{SO}$ distance are always present in the automatic pipeline inspection and it is important to ensure that the $d_{SO}$ distance stays within a known range and the effect of the β angle on the echoes received for the estimation of the thicknesses should be characterized.

## 3. Materials and Methods

For the novel method proposed to design the sensor carrier, we first characterized the blind spot and the behavior of the ultrasound beam under controlled conditions, with the aim of determining the effect of the β angle and the $d_{SO}$ distance between the transducer and the specimen. Later, we performed the mechanical analysis to propose the geometric distribution of the transducers in the sensor carrier to ensure that echo capture was achieved successfully for straight, concave, and convex pipeline sections. In the third stage of the design, we performed the simulation to ensure that the sensor carrier adapts to the pipes for different curvatures, keeping the wheels in contact with the inner wall. In the last stage, we improved the online signal reduction technique [38], that we used to compress the ultrasound signals. This improvement allows compensation of the attenuation caused by the increase in distance.

### 3.1. Ultrasound Beam Characterization for Pipeline Inspection

**Figure 3:**
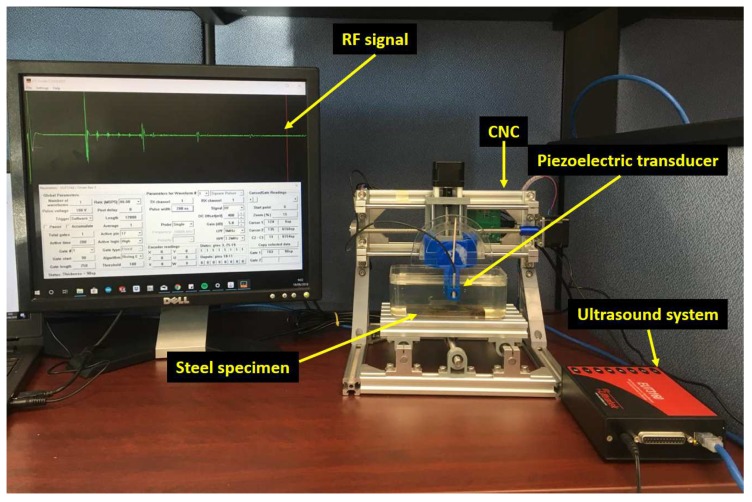
Experimental setup to study the behavior of the ultrasound beam for the determination of the effects of the β angle and the $d_{SO}$ distance between the transducer and the steel specimen.

Figure 3 shows the experimental setup for the ultrasound beam characterization. For the test, we used one flat focus-type immersion transducer of 5-MHz piezoelectric-type (Technisonic Research Inc., Fairfield, CT, USA), with a diameter of 7.11 mm (0.280 in) and a focal length of 33.02 mm (1.3 in). The transducer was customized for pipeline inspection and manufactured according to the standard ASTM E-1065; therefore, for the same application, other transducer models have similar conditions. The couplant was water, and we assumed its acoustic velocity to be 1480 m/s. If the couplant medium changes, only the ultrasonic velocity in the analysis should be changed. The material to inspect was 1020 carbon steel with an acoustic velocity of 5890 m/s. The material was a plate section with a $t_{m}$ thickness of 9.3 mm ± 0.2 mm.

We used the woodpecker CNC machine model 2.6 PCB to control the $d_{SO}$ distance between the transducer and the specimen. In the *z*-axis, the CNC has a displacement resolution of 0.1 mm. We varied the β angle with a 3D component for coupling the CNC and the middle-point of the transducer. To measure the β angle, we used a protractor considering an accuracy of ±0.5 degrees. We began the test with a $d_{SO}$ distance of 1 mm, and we increased it by increments of 1 mm until reaching 10 mm. Next, we increased the $d_{SO}$ distance in increments of 2.5 mm until reaching 40 mm. For each $d_{SO}$ distance, we captured data for varying values of β, starting at 0 degrees and increasing in units of 1 degree until no outer pipe wall echo was received. The instrument used for the data acquisition was the EUT3160 (Ultratek, Santa Clara, CA, USA) with the following parameters: a pulse voltage of 100 V with a pulse width of 200 ns, a receiver gain of 5 dB, a low pass filter of 9 MHz, a high pass filter of 1.2 MHz, a resolution of 8-bit, and a sampling rate of 80 MHz.

**Figure 4:**
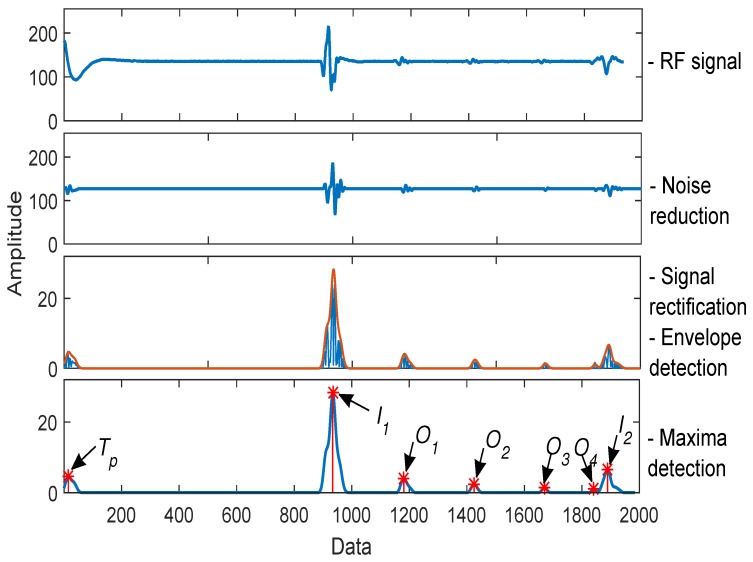
Ultrasound signal processing technique steps: noise reduction, signal rectification, envelope detection, and maxima detection [38].

For the signal processing, we used the technique proposed by Soto et al. [38]. The technique is composed of four steps: noise reduction, signal rectification, envelope detection, and maxima detection (see Figure 4). The noise reduction step is very useful in noisy environments and practical applications which have a high sampling frequency. For example, in medical ultrasound B-scans, this step helps to reject the small echoes from backscattering that blur the tissue interface and reduce the image contrast [41,42]. In the automatic inspection of pipes, the noise reduction step helps to detect small thick-walled pipe defects; because the amplitude of the echo signals drowns in the noise, this affects the extraction of the defect signal and its position [43]. For the first step (noise reduction), we used a finite impulse response (FIR) bandpass filter. Equation (2) shows the mathematical filter structure, where N−1 is the order filter, *N* is the coefficient number, $b_{k}$ is the filter coefficient, x(n) is the ultrasound RF signal, and $y_{1}\left(n\right)$ is the output signal:

(2) $$y_{1}\left(n\right)=\sum_{k=0}^{N-1}b_{k}\;x\left(n-k\right).$$

We used the Hamming window technique to compute the coefficients. The filter must be of the bandpass type, so, for the estimation of the coefficients, we used a cutoff frequency for the high pass of 4.5 MHz and a cutoff frequency of 5.5 MHz for the low pass. The number of coefficients used was 32 because it is the minimum coefficient quantity suggested by Soto et al. [38]. In the second step, we performed the signal rectification with a midpoint of 127 due to the capture of the data being performed in 8 bits. Along with the rectification, we applied a threshold to accept data with a significance greater than 2 and to reject data with a significance lower or equal than 2, this type of threshold filter is frequently used with ultrasound signals. Equation (3) shows the decision rule for the rectified value:

(3) $$y_{2}\left(n\right)=\left\{\begin{array}{cc} \left|y1(n)-127\right|, & if\;\left|y1(n)-127\right|>2,\\ 0, & if\;\left|y1(n)-127\right|\leq 2. \end{array}\right.$$

In the third step of envelope detection, we used an FIR-type low pass filter. Equation (4) shows the mathematical filter structure, where M−1 is the filter order, *M* is the coefficient number, $c_{m}$ is the filter coefficient, $y_{2}\left(n\right)$ is the rectified signal with reduced noise, and $y_{3}\left(n\right)$ is the output signal:

(4) $$y_{3}\left(n\right)=\sum_{m=0}^{M-1}c_{m}\;y_{2}\left(n-m\right).$$

We used the Hamming window technique to compute the $c_{m}$ coefficients. The number of coefficients used was 32. The cutoff frequency was of 1 MHz. Finally, in the fourth step of maximum detection, we used the criteria of the first derivative to find the maximum of each envelope. Figure 4 graphically shows the signal processing in every one of the four steps for an RF ultrasound signal of 2000 data, with a $d_{SO}$ distance of 10 mm, and a β angle of 0 degrees. Figure 4 shows how noise reduction facilitates echo analysis. Subsequently, following signal rectification, an envelope signal is obtained to detect the maxima. The maxima detection step in Figure 4 shows the position of the maximum of $T_{p}$, $I_{1}$, $O_{1}$, $O_{2}$, $O_{3}$, $O_{4}$, and $I_{2}$, explained in Figure 2.

**Figure 5:**
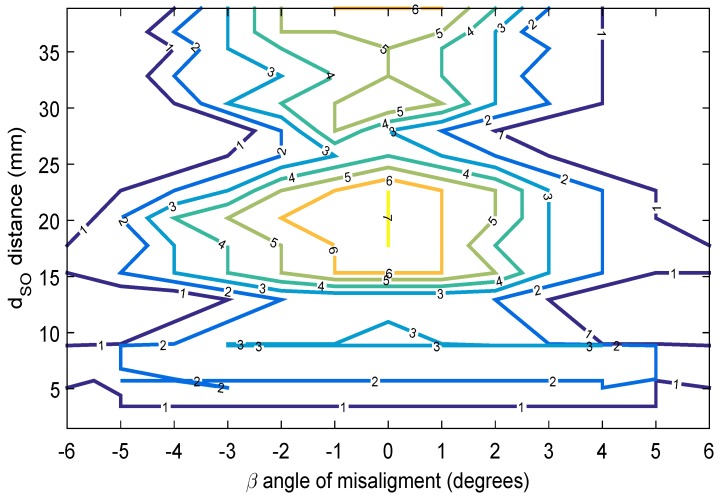
Contour graph that shows the number of $O_{n}$ echoes between the echoes $I_{1}$ and $I_{2}$, varying the $d_{SO}$ distance and the β angle.

Figure 5 shows the contour graph, which represents the behavior of the $O_{n}$ echoes between the internal wall echoes $I_{1}$ and $I_{2}$, when the $d_{SO}$ distance varies between 0 and 40 mm and the β angle varies between −6 and 6 degrees. For the test, there were no echoes with a significant amplitude when the β angle was greater than 6 degrees and lower than −6 degrees. At the end of the signal processing, the only remaining values in the *x*-axis are the data maximum and its amplitude. It must be ensured that there is no overlap of echoes, as this produces greater uncertainty in the measurements made. The measurement error in the envelope detection step may be caused by thus overlapping since it estimates a maximum of a set of points accumulated locally. To avoid the superimposition of the internal wall echoes ($I_{1}$ and $I_{2}$) with the $O_{n}$ echoes, the $d_{SO}$ distance must be at least 6 mm (see Equation (1)). Figure 5 shows the initial characterization at 3 mm; however, below 6 mm, there is an overlap, which affects the thickness estimation. The PIG excites the transducers alternately to avoid significant adverse effects such as a guided wave effect or that the diffuse beam reaches the side wall. In this way, the couplant dissolves the ultrasound waves generated by a transducer before the excitation of another transducer nearby.

Figure 5 shows important information for the design of straight beam ultrasound sensor carriers; since the contour graph corroborates the claim that the $d_{SO}$ distance of 20 mm is the most effective, acquiring up to seven exterior wall $O_{n}$ echoes between $I_{1}$ and $I_{2}$ [12]. Figure 5 extends the analysis, showing the regions where echo acquisition can be guaranteed, and where the echoes can be lost due to the transducer rotation angle β (blind spot); for example, if the transducer has a β angle of ±5 degrees and a $d_{SO}$ distance of 12 mm, then not even an $O_{1}$ echo will be received, so it will not be possible to estimate the thickness of a point. On the contrary, if the transducer is at a $d_{SO}$ distance of 15 mm, and the β angle changes to ±6 degrees, it is possible to acquire external wall $O_{n}$ echoes.

Figure 5 shows the ultrasound beam characterization of the specific transducer for this application. We recommend carrying out this characterization for the inspection application when a batch of transducers is changed, since although all the transducers comply with international standards, small changes in gain or bandwidth can reduce the range of degrees at which data can be acquired. Once we had the ultrasound beam characterization, then it was possible to propose a $d_{SO}$ distance at which the transducers of the pipe wall will be positioned; we propose this $d_{SO}$ distance to be 15 mm. The advantage of selecting this $d_{SO}$ distance is that if the sensor carrier is detached from the inner pipe wall, then it will be in a region where there is still a good margin for the β angle; for example, according to Figure 5, if the transducer is positioned at a $d_{SO}$ distance of 15 mm on the sensor carrier, and, because of chattering, it moves 5 mm away from the inner pipe wall, then the transducer has a β margin of ±5 degrees. Because our sensor carrier has wheels, the $d_{SO}$ distance is never reduced in straight pipe sections, it can only be reduced when the sensor carrier runs through the convex pipeline section. The analysis and effect of the reduction of the $d_{SO}$ distance by the convex pipeline section is presented in the mechanical design section. We recommend that, if this effect is significant (more than 2 mm), the $d_{SO}$ distance should be readjusted for the straight section. Comparing that the transducer originally had a $d_{SO}$ distance of 20 mm, where if it moves away 5 mm from the inner pipe wall, then it would be at a distance of 25 mm, where, according to the contour graph of Figure 5, the β angle could not be more than 3 degrees, since the echoes would not be received.

### 3.2. Mechanical Design

**Figure 6:**
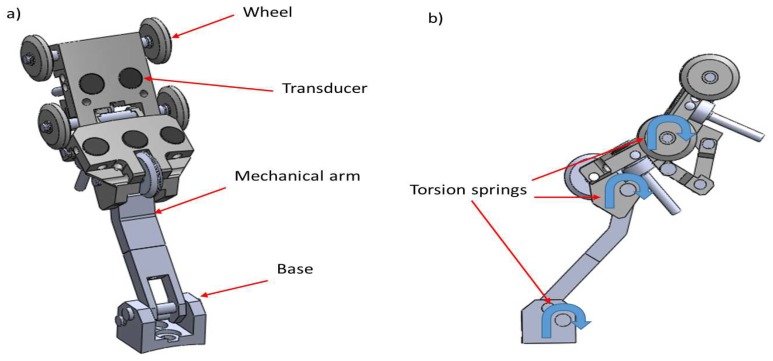
Articulated sensor carrier with piezoelectric transducers. (**a**) isometric view; (**b**) lateral view.

After determining the $d_{SO}$ distance for the transducers, the next step was to establish the rules of the mechanical design. Figure 6 shows our proposed articulated mechanism; it has a base that fixes the sensor carrier within the PIG body and a mechanical arm that facilitates the introduction to the pipeline without sticking. Five wheels were positioned so as to maintain the stability of the transducers and to ensure a constant separation from the inner pipe wall (the ideal $d_{SO}$ distance). The wheels avoid friction between the transducers and the inner wall, preventing accumulation of rust and dirt along the inspection axis, as well as shocks against protuberances. We used torsion springs to force the sensor carrier wheels to stay in contact with the inner pipe wall. The mechanical design covered the distribution of the transducers on the sensor carrier and the analysis of the wheel base length (*l*). To achieve this, we divided the design in the cross section and the longitudinal section planes.

#### 3.2.1. Cross Section Plane

**Figure 7:**
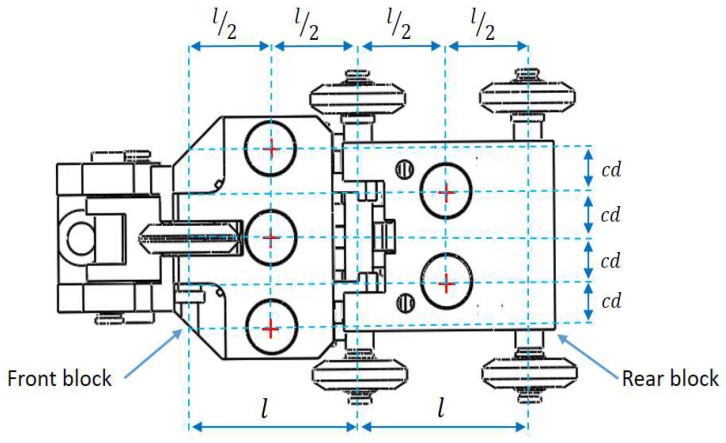
Distribution of ultrasound transducers in front and rear blocks.

Sensor carriers are designed for a specific conventional pipe diameter. The design presented in this paper is for a 254 mm (10-inch) pipe diameter, and the steps for the sensor carrier design are similar for different diameters because the only primary design criterium is the circumferential resolution (cr) of 8 mm according to international standards. The main advantage of our novel method to design the sensor carrier is that the sensor carrier has the ability to inspect concave and convex pipeline sections, geometrically adjusting itself to ensure the acquisition of the straight beam echoes. Our sensor carrier is composed of two blocks, the rear block and the front block (see Figure 7). The wheel base length of both blocks is the same (denoted as *l*). In both cases (front and rear), the transducers must be centered between the axes of the wheels (wheel base). The reason for this design consideration and the length of *l* are explained in detail in the longitudinal section analysis.

**Figure 8:**
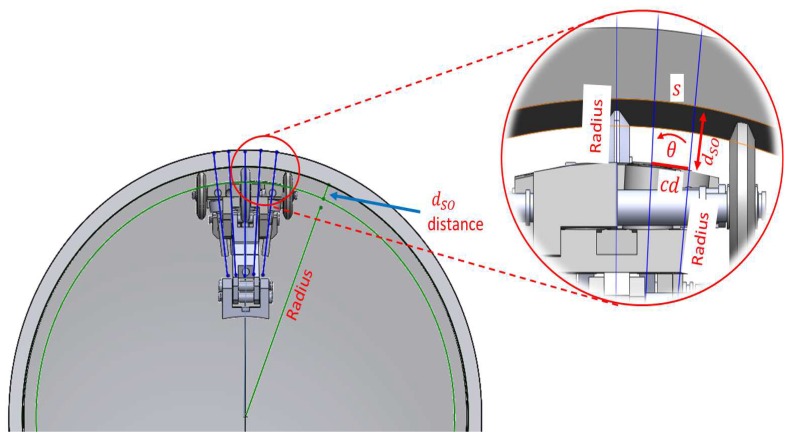
Alignment of holes with the radius of the pipe for mounting the transducers and ensuring the perpendicularity with the inner pipe wall.

Each sensor carrier has five transducers mounted; three transducers are placed in the front block and two transducers are placed in the rear block. The holes for mounting the five transducers are aligned with the pipe radius (*r*) to ensure that they are perpendicular with the inner pipe wall (see Figure 8).

International standards established in Equation (5) determine the minimum number of transducers (nt) needed to inspect a pipeline with an ultrasonic straight beam, where *d* is the pipeline diameter, and cr is the circumferential resolution of 8 mm:

(5) $$nt=\frac{\pi \;d}{cr}.$$

For example, in this case, the pipeline diameter *d* is 254 mm (10-inch), so the number of transducers (nt) should be at least 100. Dividing the 360 degrees of the circumference by the 100 transducers (nt) should give us a θ angle of no greater than 3.6 degrees (see Figure 8). However, as the inner pipe wall perimeter is 797.96 mm, there should be 100 transducers (each one with a diameter of 7.11 mm), so that the circumferential space remaining between each transducer is minimal. Therefore, the transducers were interleaved in the two blocks of the sensor carrier. This decision in the mechanical design process allowed us to satisfy the circumferential resolution (cr) without compromising the fragility of the mechanical components. The center distance (cd) of two consecutive transducers is represented by Equation (6), where *r* is the radius of the pipe:

(6) $$cd=\left(r-d_{SO}\right)\sqrt{2}\left(1-cos\left(\theta \right)\right).$$

Equation (6) was derived from the law of cosines for the isosceles triangle formed between the center of two consecutive transducers. Equation (6) has two advantages for the design of the sensor carrier: the first is that it considers the $d_{SO}$ distance and the second is that it allows us to calculate the value of cd from the number of transducers installed, which are obtained by the analysis of the θ angle.

#### 3.2.2. Longitudinal Section Plane

**Figure 9:**
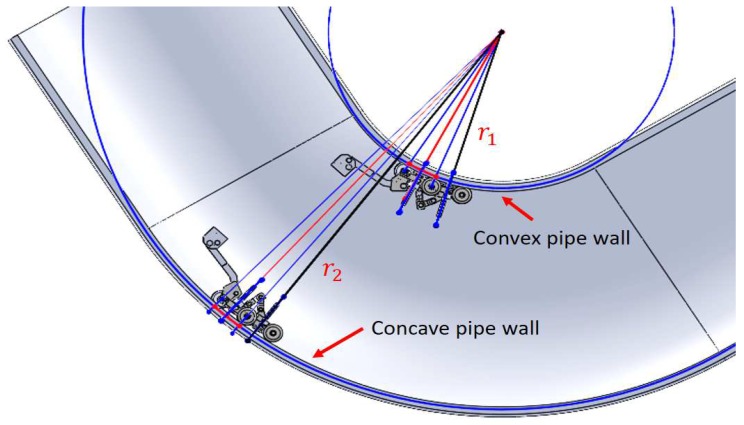
Sensor carriers inside concave and convex pipe elbow surfaces.

**Figure 10:**
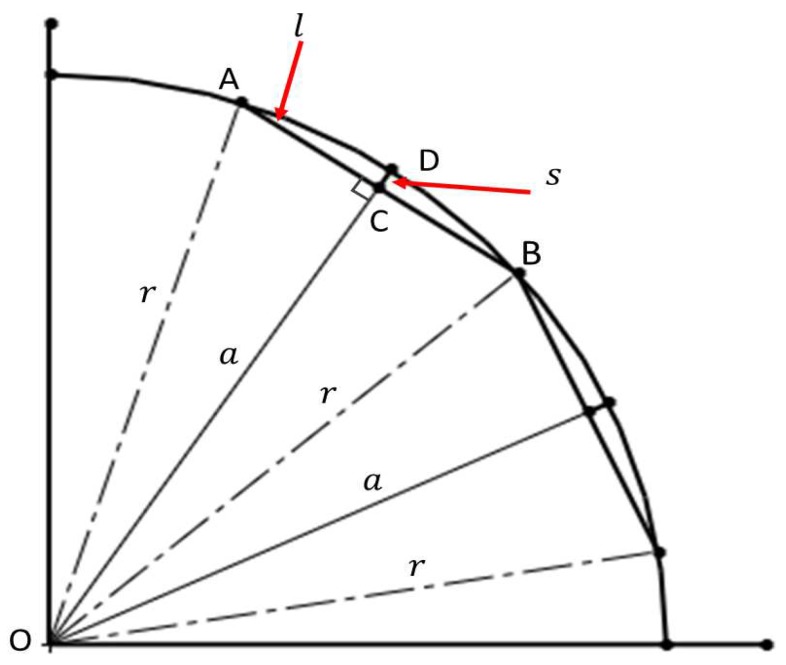
Geometric representation of a sensor carrier inside a concave curvature.

For the analysis of the longitudinal section, the sensor carrier does not have any problem if it crosses through straight pipeline sections. However, if the sensor carrier crosses sections of curved pipeline as pipe elbows (see Figure 9), then it is necessary to determine the position of the transducers in the blocks to ensure perpendicularity at any given time, and to analyze the relationship between the *l* wheel base length of the blocks and the *s* sagitta created (see Figure 10). We used the next theorem to determine the position of the transducers in the blocks: if a *r* radius is perpendicular to a chord (AB), then it bisects the chord, and bisects the main arc subtended by the chord (see Figure 10). Therefore, the transducers must be placed at point C in Figure 10, where A and B are the inner wall contact points of the wheels. In our sensor carrier, point C is denoted as l/2 and is shown in Figure 7.

The last point that we considered in the longitudinal design was the relationship between the *l* wheel base length of the blocks and the *s* sagitta created in concave and convex pipeline sections. In this case, the $d_{SO}$ distance between the transducer and the inner pipe wall increases, since a sagitta is added to the initial distance, which is established by the radius of the wheels. In sections of concave pipe, there is a possibility that the transducer will be at a distance where the β rotation angle has very little margin; for example at a $d_{SO}$ distance of 28 mm (see Figure 5). Looking at Figure 10, it is possible to obtain the relationship between the *l* wheel base length of the front and rear blocks and the *s* sagitta. The *r* radius can be expressed in Equation (7), which is derived from the rectangular triangle formed by *OCA* points:

(7) $$r^{2}=a^{2}+{\left(\frac{l}{2}\right)}^{2}.$$

In Equation (7), *a* is the apothem, and *l* is the chord from A to B. Rearranging for *l* in Equation (8) gives:

(8) $$l=2\sqrt{r^{2}-a^{2}}.$$

Considering that the apothem *a* is equal to r−s, the relationship between the wheel base length *l* of the blocks and the *s* distance in concave pipe sections is described by Equation (9):

(9) $$l=\sqrt{8\;r\;s-4\;s^{2}}.$$

For example, with a pipeline radius *r* of 254 mm, and a sagitta *s* of 2 mm, *l* must be of 44.89 mm in length. Equation (9) is important, since it allows us to derive the relationship between the *l* wheel base length of the blocks and the final $d_{SO}$ distance between the transducer and the inner pipe wall when the PIG runs through sections of concave and convex pipe.

### 3.3. Ensuring the Sensor Carrier Adaptation to Pipes of Different Curvatures

**Figure 11:**
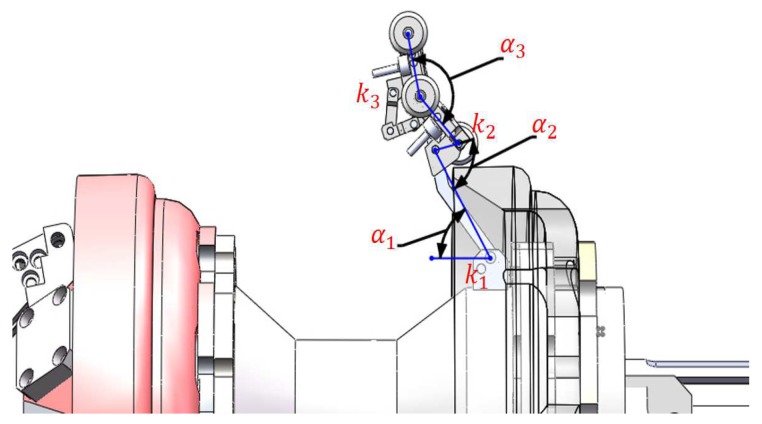
Sensor carrier in its free position.

Figure 11 shows the sensor carrier in its free position, the positions of torsion springs ($k_{1}$, $k_{2}$, and $k_{3}$), and their reference angles ($\alpha_{1}$, $\alpha_{2}$, and $\alpha_{3}$). It is necessary to ensure that the front and rear block are in constant contact with the inner pipe wall when the PIG runs. The three torsion springs are responsible for maintaining this condition. We used SolidWorks analysis tools (2016, Dassault Systemes) to simulate the PIG running in a straight pipe, and in a pipe elbow with a curvature of 1.5 *D*, where *D* is the pipeline diameter of 254 mm. The angles shown in Table 1 were calculated from the simulation and they are crucial to ensure the contact of the five wheels with the inner pipe wall. Table 1 shows the torsion spring parameters, which were obtained by a brute-force algorithm to achieve the angles desired. The torque values in Table 1 ensure contact between the wheels and the inner pipe wall because, if some torques were zero during the simulation, it would imply that some carrier block is loose.

### 3.4. Improvement of the Signal Reduction Technique

**Figure 12:**
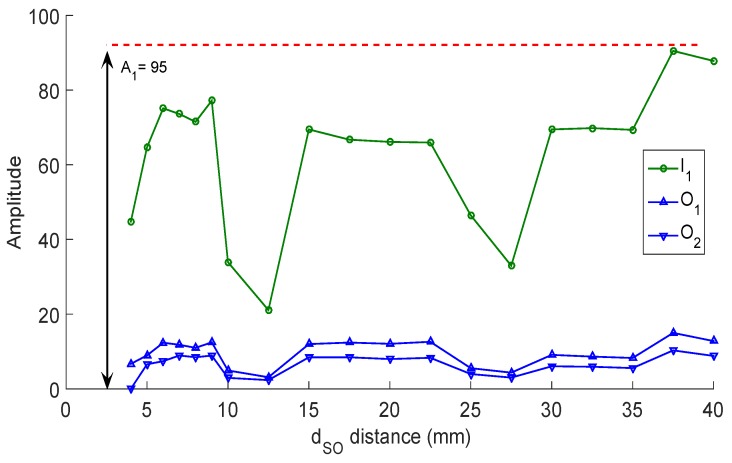
Amplitude relationship for echoes $I_{1}$, $O_{1}$, and $O_{2}$ for an excitation voltage of 100 V, using the online reduction technique [38], and varying the $d_{SO}$ distance.

The misalignment characterization between the internal pipe wall and the transducer also allowed us to analyze the echo amplitude changes when the $d_{SO}$ distance changes. Figure 12 shows these amplitude changes (for $I_{1}$, $O_{1}$, and $O_{2}$ when β is 0 degrees) after applying the reduction technique to the ultrasound signals. Figure 12 shows that $O_{1}$ and $O_{2}$ have a better amplitude at intervals of the $d_{SO}$ distance and one of these begins at 15 mm; this behavior reinforces our proposal to change the $d_{SO}$ distance to 15 mm.

It is possible to use a variable gain *k* to amplify the ultrasound signals online and compensate the attenuation of the sound pressure. However, in the PIG, the dynamic adjustment of the gains for the FIR filters complicates the defect size estimation. The better solution is to find a fixed *k* gain value for each one of the FIR filters in the online reduction technique [38]. This type of solution is already used for the transducer excitation voltage, where a voltage is fixed to reduce the error in the defect size estimation. For our system, we use a fixed value of 100 V for the excitation voltage [40], and, at the signal processing level, we propose to improve the online reduction technique, changing the function of the FIR filters. The improvement consists of improving the tuning of the bandpass and lowpass filters so that they not only filter but also amplify, with a gain $G_{1}$ for the bandpass filter and a gain $G_{2}$ for the lowpass filter.

**Figure 13:**
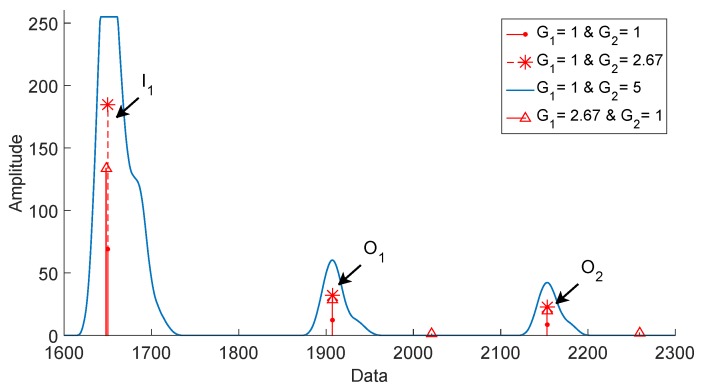
Analysis of the effects on the signal processing when the gains of the bandpass ($G_{1}$) and lowpass ($G_{2}$) filters change; at an excitation voltage of 100 V and a $d_{SO}$ distance of 15 mm.

Figure 12 shows that the maximum value of the echoes $I_{1}$ ($A_{1}$). We propose an adjustment of the form $k=A_{2}/A_{1}$, where *k* is the gain and $A_{2}$ is the desired amplitude. The *k* gain must be accurately estimated because, if it is exceeds the true value of *k*, the envelope is saturated or if the *k* gain is not well distributed between the filters; then, the measurements can be affected. The amplitude $A_{2}$ should have a value of 254 since this value is the limit, expressed in 8 bits, and does not alter the envelope construction. It is important to mention that proposing a higher amplitude of 254 for $A_{2}$ drastically increases the amount of data to be stored [39]. Dividing $A_{2}$ by $A_{1}$, we obtain a *k* gain value of 2.67; this gain is assigned to $G_{1}$ and $G_{2}$. We used the Hamming window technique to compute the filter coefficients (explained in Section 3.1). Figure 13 shows the effects of changing $G_{1}$ and $G_{2}$ for the reduction technique. We tested the system with $G_{1}=1$ and $G_{2}=5$ to show the problem of the channel saturation for the envelope construction because it was saturated, losing the position of the maximum for the echo $I_{1}$. When $G_{1}$ was changed to 2.67, the position of $I_{1}$ was displaced. This affects the thickness measurement, so the bandpass filter should not be modified. When $G_{2}$ was equal to 2.67, the echoes were amplified correctly so that the lowpass filter could be modified. In this way, our improvement in the signal processing level was to find the fixed gains for the two FIR filters, with $G_{1}=1$ and $G_{2}=2.67$ Thanks to the added step in the design of the sensor carrier, it was possible to ensure that the capture of the ultrasound echoes compensates for the attenuation of the sound pressure without saturating the signals or generating more information to be stored.

## 4. Results

We grouped our tests into three stages. In the first stage, we determined the work area of the sensor carrier, when it moves through sections of concave and convex pipes. In the second stage, we tested the behavior of the sensor carrier for a straight pipe section. In this test, we compared the configuration of our sensor carrier against others to demonstrate the advantages of our proposed change in the $d_{SO}$ distance. In the third stage, we tested the behavior of the sensor carrier inside a pipe elbow. The goal of this test was to show the ability of the sensor carrier to adapt with the shape of the pipe elbow and its ability to improve the echo acquisition on those surfaces.

### 4.1. Obtaining the Work Area of the Sensor Carrier

**Figure 14:**
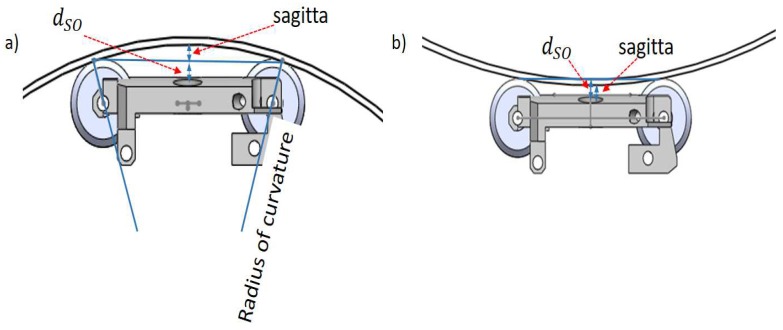
Longitudinal section plane. (**a**) sensor carrier on a concave surface; (**b**) sensor carrier on a convex surface.

In the previous section, we determined that the pipeline diameter would be 254 mm, and we proposed to use 100 transducers to satisfy the circumferential resolution (cr) of 8 mm. When the PIG crosses the pipe elbows, some sensor carriers must adapt to concave surfaces and others to convex surfaces (see Figure 9). In the pipe elbows, the $d_{SO}$ distance increases when the sensor carrier crosses through the concave pipe wall (see Figure 9, radius r2). Figure 14a shows the sagitta formed in the concave pipe wall. The $d_{SO}$ distance decreases when the sensor carrier crosses through the convex pipe wall of the pipe elbow (see Figure 9, radius r1). Figure 14b shows the sagitta that becomes the new $d_{SO}$ on the convex surface.

**Figure 15:**
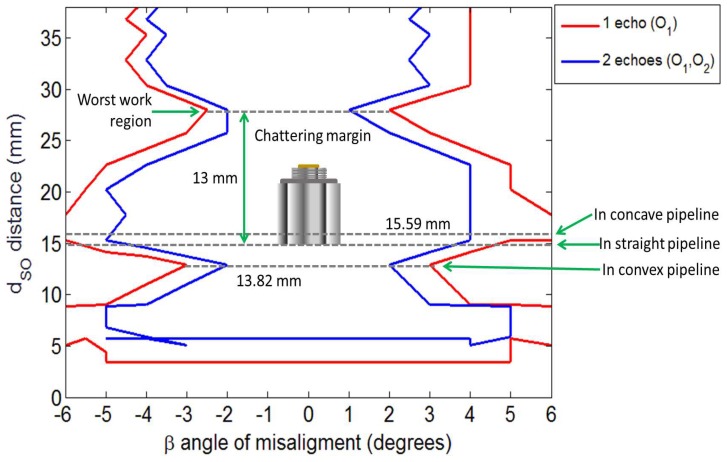
Transducer work area with the parameters of our sensor carrier.

The size of the sagitta depends on the curvature selected for the pipe elbows, and the commercial curvatures are 1.5 D, 3.0 D, and 5.0 D, where D represents the diameter of the pipe to be inspected. The analysis presented is for pipe elbows with a curvature of 1.5 D, since it is one of the most extreme curvature, maximizing the concave and convex sections. The radius of curvature of the concave surface is 2.0 D, and the radius of curvature of the convex surface is 1.0 D. We used a wheel base (*l*) of 33 mm in length, and a $d_{SO}$ distance of 15 mm. Using Equation (9) and the radius (1.0 D and 2.0 D), we obtained the sagittas for the concave and convex sections. Table 2 shows the design parameters of our sensor carrier. The wheel base (*l*) can be changed according to criteria of the designer, but its effect on the sagitta should be taken into account. Figure 15 shows the final work area of the sensor carrier when it is passing through straight, concave and convex pipe sections.

Figure 15 shows the advantages of the method improvements that we proposed in this paper. When the sensor carrier runs through convex surfaces, the transducer will be as close as possible to the pipe, with a distance of 13.82 mm. With this limited distance, there is a little margin of transducer rotation, but this rotation angle β would be minimal since the transducer would be attached to the inner pipe wall. Figure 15 also shows that, when the transducer runs through concave surfaces, it leaves a good $d_{SO}$ distance of 15.59 mm since the rotation angle β can be up to 6 degrees, and the transducer can receive at least one external wall echo. Figure 15 also shows that, with a distance of 15 mm, a margin of approximately 13 mm is needed to reach the region where the $d_{SO}$ distance has the highest value, as the rotation angle β is minimal. Other authors [12] suggested a distance of 20 mm, taking into account only the number of external wall echoes received; however, with this distance, the transducer could only detach 8 mm before reaching the least effective distance. Our analysis allows us to ensure that the transducer is at a distance where not many echoes are received, but it is possible to measure echoes easily. There is no reduction in the measurement accuracy, since the ultrasound system would capture six $O_{n}$ echoes (see Figure 5), only one less echo than with a $d_{SO}$ distance of 20 mm, improving the rotation margin and obtaining the echoes $I_{1}$, $O_{1}$, and $O_{2}$ with a good amplitude (see Figure 12). The transducers have a 6-degree rotation margin, and the transducer can be moved up to 13 mm to reach the worst work region.

### 4.2. Inspecting a Straight Pipe Section

**Figure 16:**
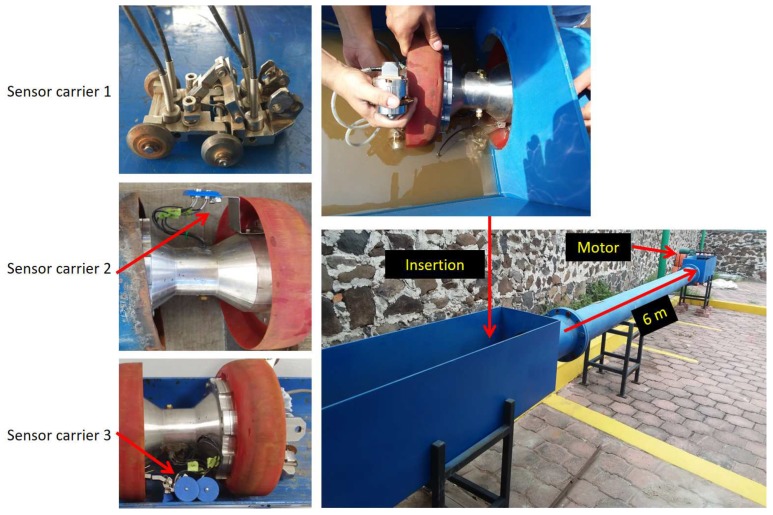
Experimental setup for straight pipe inspection with three different sensor carrier configurations.

In the second stage, we tested the behavior of the sensor carrier for a straight pipe section. In this test, we compared the configuration of our sensor carrier against others to demonstrate the advantages of our design. Figure 16 shows the experimental setup. The pipe section consisted of six meters of carbon steel pipe with a nominal thickness of 9.27 mm. The displacement was measured through a servomotor with an approximate linear speed of 10 cm/s. For the test, we used three different sensor carriers. Sensor carrier one had the same design parameters shown as in Table 2, except the $d_{SO}$ distance, which was changed to 6 mm. We calculated its torsion springs with the same methodology of the Section 3.3. Sensor carrier two considered a $d_{SO}$ distance of 20 mm, with a rigid arm. Sensor carrier three was our proposed design, and its parameters are described in Table 2. The data was captured at intervals of 5 mm.

We used two metrics to compare the results obtained by the three sensor carriers. The first metric was the mean squared error (MSE). Equation (10) shows the computation of the MSE, where *n* is the data number, and $\hat{Y_{i}}$ is the estimated thickness:

(10) $$MSE=\frac{1}{n}\sum_{i=1}^{n}{\left(Y_{i}-\hat{Y_{i}}\right)}^{2}.$$

**Figure 17:**
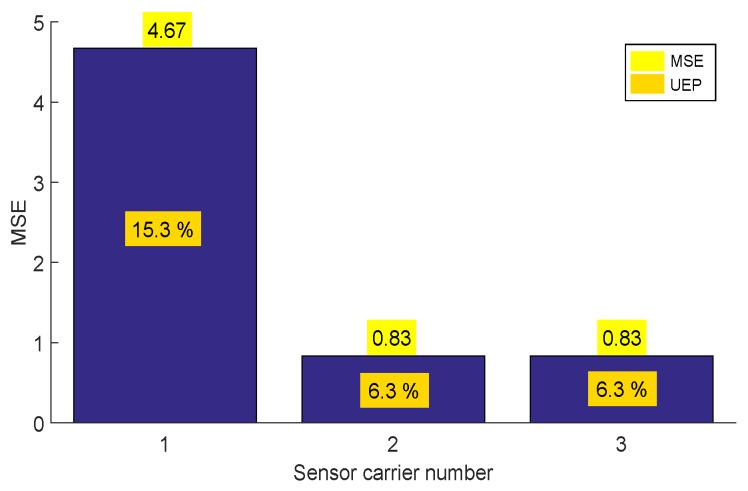
Mean squared error (MSE) and undetected echoes percentage (UEP) of the three sensor carriers for straight pipe inspection.

The estimated thickness used for the test was the nominal thickness of the pipe of 9.27 mm. We used this thickness as the estimated thickness since, under real conditions, it is very complicated to ensure that the transducers pass through the same points and to compare the results among themselves. We used an encoder as the trigger to capture data at intervals of 5 mm, and it was not possible to estimate the thicknesses due to the position of the sensor carriers. The second metric used to measure this problem is the undetected echoes percentage (denoted as UEP), which is useful to compare the type of sensor carriers versus the portion of signals captured without echoes $O_{n}$. Figure 17 shows the MSE bar graph and the UEP for the different sensor carriers. This test helped us to demonstrate that it is not only the installation of wheels or joints that can improve the wall thickness measurement.

### 4.3. Inspecting an Pipe Elbow for Concave and Convex Surfaces

**Figure 18:**
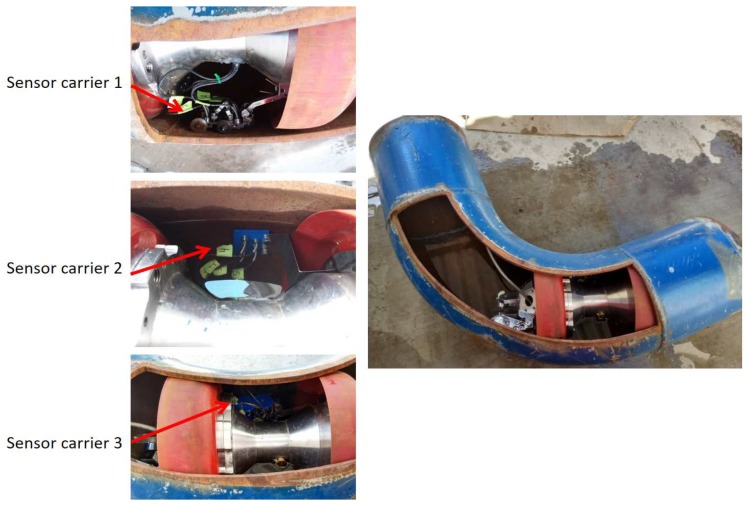
Experimental setup for concave and convex surfaces inspection with three different sensor carrier configurations.

Figure 18 shows the pipe elbow used to test the behavior of the sensor carrier for concave and convex surfaces. The pipe elbow had a diameter of 1.5 D carbon steel with a nominal thickness of 9.27 mm. The displacement was achieved manually. For the test, we used the three different sensor carriers again, but, with two runs, the first run for the concave part of the pipe elbow and the second run for the convex part.

**Figure 19:**
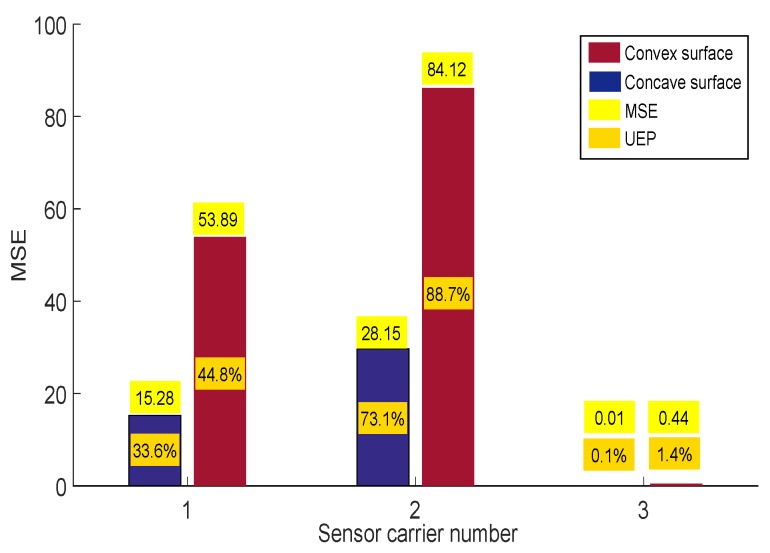
Mean squared error (MSE) and undetected echoes percentage (UEP) of the three sensor carriers for concave and convex surface inspection.

Figure 19 shows the MSE bar graph and the UEP for the different sensor carriers. The result confirms that our mechanical design for the sensor carrier adapts more effectively to the two types of surfaces. Figure 19 also shows that the rigid sensor carrier (number 2) increased its MSE and UEP when traveling through concave and convex surfaces.

## 5. Discussion

The ultrasonic pulse echo method allows for technicians measuring thicknesses at high speed and detect internal and external anomalies. The measurement of thicknesses for straight pipes by ultrasound is complicated when it becomes automated and under few controlled conditions. The PIG geometry, the pipeline deformations, and the girth welds cause a continuous chattering when the PIG is running, resulting in a loss of the transducers perpendicularity with the inspection points. With small misalignments of the transducers, measurements are lost and they can no longer be recovered since the PIG continues to move inside the pipeline. The search for better subsystems for PIGs aims to improve the quality and quantity of information acquired by the sensors. Improvements are commonly made at the software level, focusing the processing techniques on the extraction of information at a very low amplitude or from very noisy signals. However, the improvements can also be mechanical, in such a way that they improve the echo capture. The challenge of mechanical improvements is that they should not add more weight to the PIG, and their results should be significant.

This paper presents a novel method to design a sensor carrier that ensures the angularity and correct distance to acquire the straight beam echoes. First, the characterization of the transducers that will be used for the inspection must be carried out. Second, the $d_{SO}$ distance must be selected to ensure the greatest possible range of rotation (β angle) from the transducer in case of chattering. In the third step, the distribution of the transducers between the different blocks is defined according to their center distance (cd). In the fourth step, the calculation of the diameter of the wheels is performed. In the fifth step, the length *l* of the blocks is validated, to ensure that the thicknesses can be measured on concave and convex surfaces. In the sixth step, the torsion springs are selected by simulation to ensure the sensor carrier adaptation to the pipes for different curvatures. Finally, in the seventh step at the signal processing level, the fixed gain $G_{2}$ in the lowpass filter is adjusted to compensate the sound pressure attenuation.

**Figure 20:**
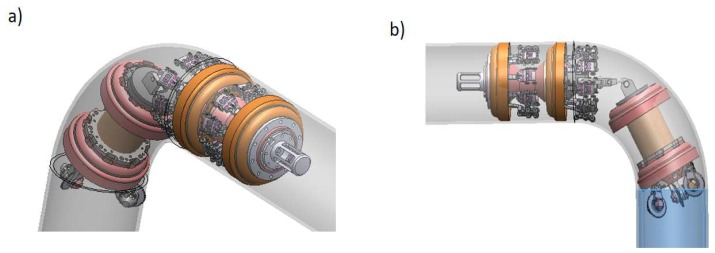
Final design for the distribution of the sensor carriers in the PIG. (**a**) isometric view; (**b**) lateral view.

Figure 20 shows the final design of the instrumented ultrasound with the set of sensor carriers for the inspection of a 254 mm (10-inch) diameter pipe. In this work, one of these mechanisms was analyzed, since all of them undergo the same inspection requirements. Each sensor carrier is mounted and radially distributed on a cylindrical capsule supported by polyurethane cups, in order to comply with the inspection resolution.

## 6. Conclusions

The improvement of technology for the PIG sub-systems aims to reduce its construction costs and increase the feasibility of data capture. In the case of PIGs instrumented with ultrasound systems for inspection, technicians seek to achieve the accurate measurement of wall thicknesses and the detection of defects without so many sensors and mechanical systems. With the reduction of costs, it becomes feasible that the inspection of pipes can be used not only for the distribution of oil, but also for other processes. In this sense, we present a novel method for the design of an articulated sensor carrier to improve the automatic pipeline inspection. To achieve this, we first analyzed the conditions of the automatic pipeline inspection, the current recommendations for state-of-the-art analysis, the different mechanical scenarios that may occur, and we added a new consideration for the design, which was the characterization of the misalignment between the inner wall pipe and the transducer.

This new consideration allowed us to change the value of the traditionally proposed $d_{SO}$ distance in state-of-the-art analysis. The change in the $d_{SO}$ distance gave a chattering margin of 13 mm in the sensor carrier against traditional designs. In addition, with the new consideration, it was possible to ensure the correct behavior of the sensor carrier when it runs inside sections of concave and convex pipelines. At the signal processing level, we found a fixed gain ($G_{2}$) in the lowpass filter to compensate the sound pressure attenuation and to obtain the echoes in a defined distance range without saturating the acquisition channels. The validation of the sensor carrier was demonstrated analytically and experimentally. The sensor carrier designed in this work was for a 254 mm diameter pipeline. It is necessary to develop sensor carriers that are compatible for a wide range of diameters; in this way, it will not be necessary to redesign them for every diameter change. However, these sensor carriers are not entirely validated for industrial applications. Due to this, we present all the design parameters for variable diameters to be inspected and build the ad hoc sensor carrier with excellent results.

The main advantages of our improvement to the ultrasound inspection are: (a) An increase in detection capacity of straight beam echoes in concave and convex pipeline sections; (b) A reduction in the risk of the PIG getting stuck in the pipeline owing to its mechanical configuration; (c) A system not actuated, so it does not consume energy from the battery system; (d) A self-adjustment for straight, concave and convex pipe surfaces; and (e) An increase in the distance margin ensuring the echo capture despite the misalignment and the variation in the distance between the transducer and the inner pipe wall.

## Figures and Tables

**Table 1:** Torsion spring parameters and their conditions for straight, concave, and convex pipe sections.

Torsion Spring	Wire Diameter (mm) ± 0.1 (mm)	Maximum Torque (N-mm) ± 0.5 (N-mm)	Reference Angle (degrees) ± 1 (degrees)	Straight Pipe		Concave Pipe		Convex Pipe	
				Angle (degrees)	Torque (N-mm)	Angle (degrees)	Torque (N-mm)	Angle (degrees)	Torque (N-mm)
$k_{1}$	3.43	4461	60	42	1631	27	1048	56	2172
$k_{2}$	2.41	1936	72	8	170	12	258	6	116
$k_{3}$	2.03	1183	150	30	22	26	145	22	123

**Table 2:** Parameters and dimension values of our sensor carrier. All parameters are expressed in mm.

Block	Total Size	Wheel Base (*l*)	Center Distance (cd)	Convex Sagitta (*s*)	Concave Sagitta (*s*)	Stand Off ($d_{\mathrm{SO}}$)	Wheel Diameter
Front	49	33	0.66	1.18	0.59	15	21
Rear	49	33	0.66	1.18	0.59	15	21
